# Appearance quality classification method of Huangguan pear under complex background based on instance segmentation and semantic segmentation

**DOI:** 10.3389/fpls.2022.914829

**Published:** 2022-10-19

**Authors:** Yuhang Zhang, Nan Shi, Hao Zhang, Jun Zhang, Xiaofei Fan, Xuesong Suo

**Affiliations:** ^1^ College of Mechanical and Electrical Engineering, Hebei Agricultural University, Baoding, China; ^2^ Key Laboratory of Microbial Diversity Research and Application of Hebei Province, College of Life Sciences, Hebei University, Baoding, China

**Keywords:** ‘Huangguan’ pear disease, deep convolutional neural networks, instance segmentation, semantic segmentation, disease severity classification

## Abstract

The ‘Huangguan’ pear disease spot detection and grading is the key to fruit processing automation. Due to the variety of individual shapes and disease spot types of ‘Huangguan’ pear. The traditional computer vision technology and pattern recognition methods have some limitations in the detection of ‘Huangguan’ pear diseases. In recent years, with the development of deep learning technology and convolutional neural network provides a new solution for the fast and accurate detection of ‘Huangguan’ pear diseases. To achieve automatic grading of ‘Huangguan’ pear appearance quality in a complex context, this study proposes an integrated framework combining instance segmentation, semantic segmentation and grading models. In the first stage, Mask R-CNN and Mask R-CNN with the introduction of the preprocessing module are used to segment ‘Huangguan’ pears from complex backgrounds. In the second stage, DeepLabV3+, UNet and PSPNet are used to segment the ‘Huangguan’ pear spots to get the spots, and the ratio of the spot pixel area to the ‘Huangguan’ pear pixel area is calculated and classified into three grades. In the third stage, the grades of ‘Huangguan’ pear are obtained using ResNet50, VGG16 and MobileNetV3. The experimental results show that the model proposed in this paper can segment the ‘Huangguan’ pear and disease spots in complex background in steps, and complete the grading of ‘Huangguan’ pear fruit disease severity. According to the experimental results. The Mask R-CNN that introduced the CLAHE preprocessing module in the first-stage instance segmentation model is the most accurate. The resulting pixel accuracy (PA) is 97.38% and the Dice coefficient is 68.08%. DeepLabV3+ is the most accurate in the second-stage semantic segmentation model. The pixel accuracy is 94.03% and the Dice coefficient is 67.25%. ResNet50 is the most accurate among the third-stage classification models. The average precision (AP) was 97.41% and the F1 (harmonic average assessment) was 95.43%.In short, it not only provides a new framework for the detection and identification of ‘Huangguan’ pear fruit diseases in complex backgrounds, but also lays a theoretical foundation for the assessment and grading of ‘Huangguan’ pear diseases.

## 1 Introduction

Pears are fruits produced and consumed around the world, growing on a tree and harvested in the Northern Hemisphere in late summer into October. The pear tree and shrub are a species of genus Pyrus, in the family Rosaceae, bearing the pomaceous fruit of the same name (Ikinci et al., 2014). Several species of pears are valued for their edible fruit and juices, while others are cultivated as trees. China is the world’s largest producer and consumer of pears, and its pear cultivation area and output rank first in the world (Oyom et al., 2022). ‘Huangguan’ pear is a mid-early mature pear variety cultivated by China. It has the advantages of large fruit size, high quality, early fruit, and good yield. It can meet the demand for high-quality pears in the fruit market. After years of demonstration and promotion, ‘Huangguan’ pear has become one of the main pear tree varieties in most regions, providing huge economic benefits for ‘Huangguan’ pear producers and exporting countries. It is worth emphasizing that the economic value of ‘Huangguan’ pear fruit depends to a large extent on the aesthetics of its appearance. The best-looking fruits are for export, the less diseased ones are reserved for domestic consumption, and the worst ones are used for further processing to make canned fruits or jams. However, the quality grading of ‘Huangguan’ pear is a time-consuming and laborious process. So far, it has almost completely relied on human inspection and manual observation of disease symptoms to judge the grade of ‘Huangguan’ pear. This method is costly and has highly subjective and low efficiency and timeliness. However, early automated grading systems have extensively utilized image processing algorithms and relied on manually defined image features to build classifiers (Suykens, 2001; Zeng et al., 2020), limiting the robustness and generalization (Xu and Mannor, 2012) of detection performance due to the variance of fruits types, appearances, and damage defects.

In recent years. With the advancement of agricultural informatization, deep learning and machine learning are widely used different areas in agriculture (Dobrota et al., 2021; Yang et al., 2022a)in particular in crop disease detection (Liu et al., 2018; Pooja et al., 2018; Yu et al., 2018; Özden, 2021; Tassis et al., 2021; Wang et al., 2021; Wang et al., 2022; Yang et al., 2022b), experts and scholars have achieved fruitful results in the research of plant disease identification. Wu et al. (2020b) used Mask R-CNN and VGG models to judge whether 6400 mango images are good or bad, with an accuracy rate of 83.6% and expressed by PCA. Ren et al. (2020) used the tomato plant diseases in the Plant Village data set and the improved VGG to propose a model that can identify tomato leaf diseases, with an accuracy rate of more than 95%.In the food industry, a model based on CNN was introduced for identification of soft-shell shrimp. The proposed model attained an average accuracy of 97% (Liu, 2020). Ireri et al. (2019) introduced a tomato grading machine vision system. The proposed system performed calyx and stalk scar detection for both defected and healthy tomatoes based on regions of interest. The radial basic function support vector machine classifier achieved 97.09% accuracy rate for healthy and defected tomatoes. Farooq and Sazonov (2017) used CNN to classify different food groups. The classification accuracy for 7 and 61 different classes was 94.01% and 70.13%, respectively. Liang et al. (2019) proposed a plant disease severity estimation network PD2SENet, which achieves excellent comprehensive performances. Lu et al. (2017) developed an application for diagnosing diseases in wheat leaves using two steps: a disease location step and a classification step. Wu et al. (2020a) proposed an automatic and efficient apple defect identification method based on laser-induced light backscatter imaging and convolutional neural network algorithm. Sofu et al. (2016) proposed an automatic apple sorting and quality inspection system that apples were sorted into different classes by their color, size and weight. It also detected apples affected by scab, stain and rot. The average grading accuracy rate is 73–96%. Wang et al. (2017) applied 5 convolutional neural networks with different structures to estimate the severity of plant diseases, and fine-tuned the existing network models using transfer learning to improve the model accuracy. The above research has used traditional machine learning or deep learning to identify plant diseases, but the refinement and generalization capabilities need to be improved. Although some progress has been made in the research of fruit disease segmentation under complex background, the research of ‘Huangguan’ pear has not made significant progress. Convolutional neural networks have led to a series of breakthroughs for image classification (He et al., 2015). This article uses a convolutional neural network (CNN) to automatically extract data features by introducing local connections, pooling and other operations. In the first step, the strength segmentation model was used to remove the background of ‘Huangguan’ pear, which ensured the fineness of the next step of grading (He et al., 2018). Then, the semantic segmentation model was used to segment the disease of ‘Huangguan’ pear, and the proportion of disease pixels in ‘Huangguan’ pear was calculated (Chen et al., 2018). Finally, by performing transfer learning on ImageNet data. The over-fitting problem caused by the small sample data domain was optimized and the grading model was used to achieve the quality grading of ‘Huangguan’ pear (Akiba et al., 2017). Experiments show that this method can not only improve the recognition accuracy of ‘Huangguan’ pear disease, but also is suitable for classification of ‘Huangguan’ pear disease images in generalized scenarios.

The main contributions of this research are as follows:

For complex background images, a two-stage segmentation model of ‘Huangguan’ pear disease based on deep learning was proposed. The model achieved accurate segmentation of ‘Huangguan’ pear and disease. It provided the basis for establishing the classification model of ‘Huangguan’ pear disease severity.
By adopting a three-stage continuous segmentation and classification method, the complementary advantages of Mask R-CNN, DeepLabV3+ and Resnet50 models are fully utilized. Compared with the single-stage model, this model has better segmentation and classification effects.
A method for grading the severity of ‘Huangguan’ pear disease was proposed. By calculating the ratio of the area of diseased spots to the area of ‘Huangguan’ pear fruit, it provides technical support for the accurate classification of the appearance quality of ‘Huangguan’ pear in actual production.
It can effectively solve the problem of inaccurate grading of ‘Huangguan’ pears caused by manual sorting, which is time-consuming and laborious and easy to distract. It provides a new idea for the automatic grading of ‘Huangguan’ pear appearance quality.

## 2 Materials and methods

### 2.1 Data set production and processing

**Figure 1:**
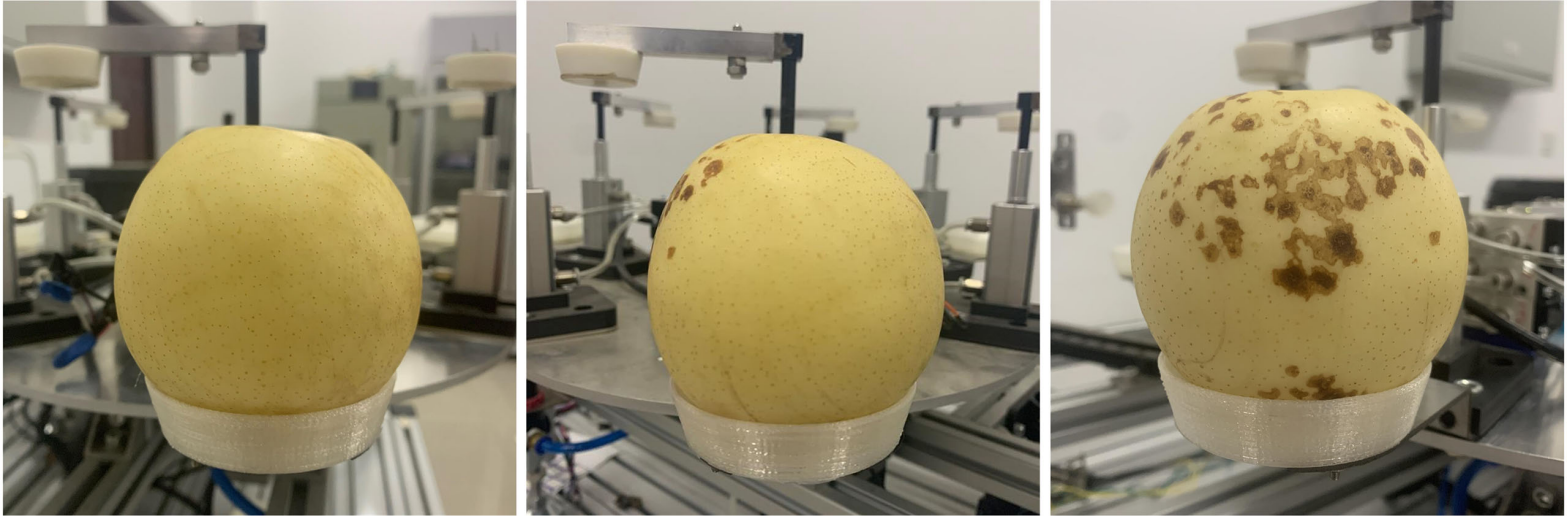
Some pictures of ‘Huangguan’ pear.

The data set used in this article has a total of 5562 images of ‘Huangguan’ pear. Taking into account the diversity of lighting conditions in practical applications, The data was collected in three different periods from July to December 2021: In the morning (8:30–10:00), noon (12:30–14:00) and afternoon (15:30–17:00) in the laboratory with camera. This leads to problems such as background noise, distance, location, and lighting conditions of ‘Huangguan’ pear. It is the existence of these problems that can improve the generalization ability of the model in different scenarios and improve the robustness of the model. Part of the ‘Huangguan’ pear image is shown in **Figure 1**. According to the ‘Huangguan’ pear samples displayed in the data set, the identification and segmentation of ‘Huangguan’ pear fruit disease mainly have the following difficulties: 1) ‘Huangguan’ pear background interferes with segmentation, and the different brightness of ‘Huangguan’ pear imaging caused by factors such as light can easily be mistaken for disease; 2) ‘Huangguan’ pear disease are irregular in shape, some are small, and the initial disease are difficult to detect with the naked eye, which increases the difficulty of disease segmentation; 3) ‘Huangguan’ pear have different shooting backgrounds, and the quality of the background processing directly affects the classification of ‘Huangguan’ pear.

### 2.2 Image data enhancement

**Figure 2:**
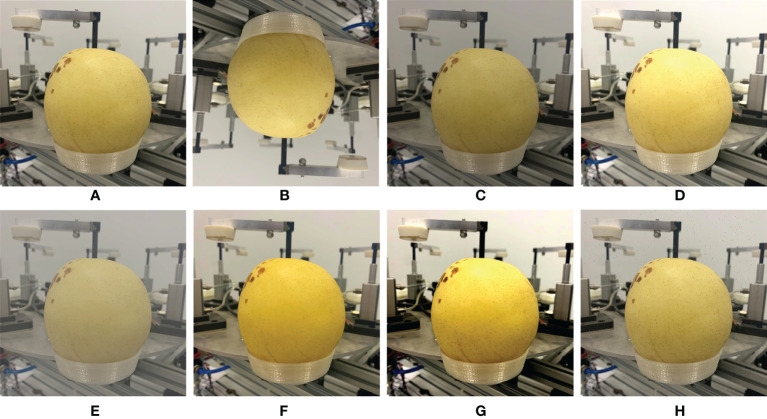
The image enhancement of **(A)** Original image, **(B)** Vertical flip, **(C)** 0.8 Brightness, **(D)** 1.2 Brightness, **(E)** 0.6 Contrast, **(F)** 1.6 Contrast, **(G)** Change chroma, and **(H)** Add salt noise.

The sample distribution of each type of disease in the data set is not uniform, and the limited training data is easy to overfit the deep learning model. In deep learning, the use of data augmentation methods to expand the data can improve the generalization ability of the model. The training data of this study uses the Image Data Generator online enhancement method under the Keras framework. That is, an enhancement method is randomly selected for each batch of data during the training process, without increasing the number of original data sets. In order to avoid changing the original data characteristics and better simulate the differences of samples under real shooting conditions, the training set of this research mainly adopts the following data enhancement methods: 1) Flip: Flip the image vertically to simulate the randomness of the shooting angle when the sample is collected, and will not change the shape of the diseased spot and the distribution of the diseased spot on the leaf. 2) Color jitter: Change the brightness of the image to randomly jitter between 0.8-1.2 times. Change the contrast of the image to randomly jitter between 0.6-1.6 times. Change the chromaticity of the image to jitter randomly between 0.7-1.4 times. Simulate lighting differences and ensure that the parameters conform to the actual shooting conditions to avoid image distortion. 3) Add noise: Add salt and pepper noise with a signal-to-noise ratio of 0.95 to the image to simulate the noise generated during the shooting process and weaken the high-frequency features to prevent the model from overfitting. The result of data enhancement is shown in **Figure 2**.

### 2.3 Labeling of diseased spots of ‘Huangguan’ pear fruit

**Figure 3:**
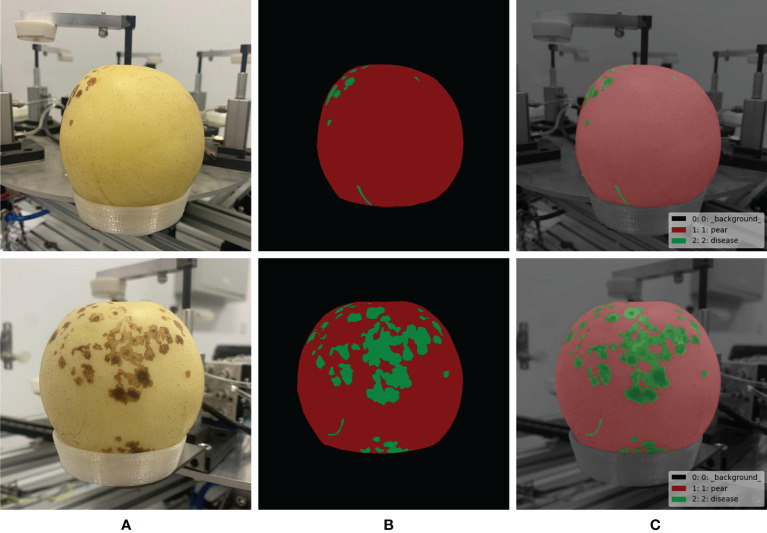
Pixel level labeling of ‘Huangguan’ pear fruit and disease spot. **(A)** Original images, **(B)** Pear-disease-labels and **(C)** Composite diagrams.

To train the disease segmentation model, the disease need to be marked as shown in **Figure 3**. The labeling of ‘Huangguan’ pear disease is time-consuming and laborious, with a large number of small targets. The finer annotations help Mask R-CNN and DeepLabV3+ to perform finer segmentation of ‘Huangguan’ pears and disease, laying the foundation for the classification of ‘Huangguan’ pears. The labeling is divided into three scenes including background, pear and diseased spots, and labeling is carried out with LabelMe (Russell et al., 2008), an image semantic segmentation labeling tool.

### 2.4 Grading method for the severity of fruit diseases of ‘Huangguan’ pear

The classification of disease severity is the basis for formulating prevention and control strategies. Three methods are usually used in practice. The first method is to calculate the ratio of the number of infected fruits per unit area to the total number of fruits. The second method is to calculate the ratio of the number of diseased fruits to the total number of fruits on the same plant. The third method is to calculate the ratio of the area of spots on the same fruit to the total area of the fruit. The third method is the basis for accurately estimating the severity of crop diseases in a region. Therefore, we used the third method, which uses the ratio of the spot area to the total area of the same fruit as the basis for classification of disease severity. This method is mainly based on the opinions and practical experience of fruit farmers who have been engaged in fruit grading for many years.

By calculating the ratio of the area of the diseased spot to the area of the fruit, the severity of the disease of ‘Huangguan’ pear was classified. Since the ‘Huangguan’ pear fruit to be divided is located in a complex background, the target ‘Huangguan’ pear fruit and diseased spots are easily confused with other similar elements, resulting in over-segmentation or under-segmentation. Therefore, it is difficult to accurately segment ‘Huangguan’ pear fruit and diseased spots at the same time using a single-stage network. In order to ensure the accuracy of disease segmentation, the ‘Huangguan’ pear fruit in the complex background should be segmented first. Therefore, this study uses a two-stage segmentation network to classify the severity of ‘Huangguan’ pear diseases, and classifies the ‘Huangguan’ pear images according to the first, second and third levels. Specific steps are as follows. In the first stage, the segmentation target is the ‘Huangguan’ pear fruit and the complex background. The mask image obtained from the test is used to extract the ‘Huangguan’ pear fruit from the complex background, so as to obtain the ‘Huangguan’ pear fruit in the simple background. In the second stage of segmentation, the diseased spots in the ‘Huangguan’ pear fruit are taken as the target, and the proportion of the diseased spots in the ‘Huangguan’ pear fruit is obtained. As the basis for the classification of disease severity of ‘Huangguan’ pear. The formula is shown in formula (1).


(1) $$P=\frac{S_{Disease}}{S_{Pear}}$$

Among them, *S*_Pear_ represents the fruit area of ‘Huangguan’ pear after segmentation; *S*_Disease_ represents the area of the disease after segmentation; *P* represents the proportion of diseased spots on ‘Huangguan’ pear fruit.

After calculating the area of ‘Huangguan’ pear by the disease, refer to the ‘Huangguan’ Pear Fruit Grade” DB 13/T 1571—2012 issued by China. According to local standards, the proportion of fruit diseases can be divided into three grades: good and bad. Among them, 0% of diseases are first-class fruits, 2% or less are second-class fruits, and diseases greater than 2% are third-class fruits.

### 2.5 Evaluation index

In order to reasonably evaluate the performance of the model, the first two segmentation stages of this study used 3 commonly used evaluation indicators: Pixel Accuracy (PA), dice and Intersection over Union (IoU). The pixel accuracy is the ratio of all correctly classified pixels to the total pixels, as shown in formula (2):


(2) $$R_{PA}=\frac{\Sigma_{i=0}^{k}p_{ii}}{\Sigma_{i=0}^{k}\Sigma_{j=0}^{k}p_{ij}}$$

In the formula, *k* is the number of categories, *p_ii_* is the number of pixels that are correctly predicted, and *p*_*ij*_ represents the number of pixels whose category *i* is predicted to be category *j*

The Dice coefficient is a function that measures the similarity of two sets, and is one of the most commonly used evaluation indicators in semantic segmentation. As shown in formula (3):


(3) $$R_{dice}=\frac{2|X\cap Y|}{\left|X|+|Y\right|}$$

Where *X* is the predicted pixel and *Y* is the ground truth.

The intersection ratio is the ratio of the intersection and union of a certain type of prediction result and the true value of the model. The intersection ratio is the most commonly used evaluation index in semantic segmentation, and the expression is shown in formula (4):


(4) $$R_{IoU}=\frac{A\cap B}{A\cup B}$$

When the value of IOU is between 0 and 1, it represents the degree of overlap of the two boxes. The higher the value, the higher the degree of overlap.

The third grading stage uses 5 evaluation indicators commonly used in grading models, recall, precision, average precision (AP), F1 score and speed. Recall is the ratio of the number of correctly detected targets to all actual targets (Equation (5)). Precision is the number of correctly detected targets in all detected targets The ratio of (Equation (6)). F1 is the harmonic average of precision and recall (Equation (7)).


(5) Recall(R)=TPTP+FN

(6) $$\mathrm{Pr}ecision(P)=\frac{TP}{TP+FP}$$

(7) F1=2×Pre×RecPre+Rec

### 2.6 Model training

The hardware configuration used for training and testing in this research is as follows: Intel(R) Core(TM) i5-10400F CPU @ 2.90GHz, 16G RAM, NVIDIA GeForce GTX 1650SUPER graphics card, 64-bit Windows 10 operating system, CUDA version 10.0 and TensorFlow version 1.13.2. In order to avoid the influence of hyperparameters on the experimental results, the hyperparameters of each network are uniformly configured. After trial and error, the following hyperparameters have been determined: The learning rate is 1e-4, the epochs is 50, and the batch size is 16. If training for more than 5 generations does not further improve the accuracy, start early stopping and stop training.

## 3 Model construction

### 3.1 Input layer

The input image is a color 3-channel image of leaf disease, and the image size is uniformly adjusted to 416x416 pixels. In order to enhance the generalization ability of the model, a data enhancement method is randomly selected during the training process to process the original image, and the normalized and standardized data is used as the input of Mask R-CNN.

### 3.2 Model Mask R-CNN

**Figure 4:**
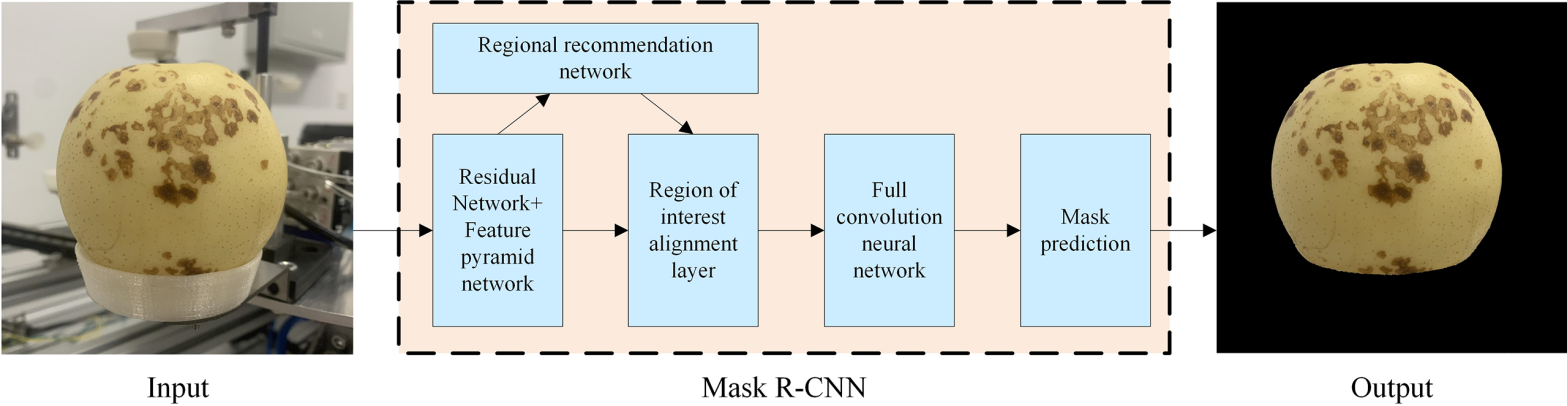
The processing flow of ‘Huangguan’ pear by Mask R-CNN network.

Mask R-CNN is a new convolutional neural network proposed by Ren et al. (2015) based on Faster R-CNN, which realizes instance segmentation. This method can not only detect the target effectively, but also complete high-quality semantic segmentation of the target. The main idea is to add a branch to the original Faster R-CNN to achieve semantic segmentation of the target. Mask R-CNN uses FPN to improve the feature extraction network, which better solves the problem of serious loss of semantic information through the feature extraction layer of FCN and SegNet (Kendall et al., 2015), and greatly improves the segmentation of small target defects. For Deeplab-v3 defect contour segmentation is not clear, Mask R-CNN replaces the interest area pooling layer with the interest area alignment layer. That is, the spatial information on the feature map is further utilized through bilinear interpolation, so as to predict a more accurate defect contour. Mask R-CNN first uses the FPN based on Resnet50 to extract the feature map of the defect image, and then uses RPN to generate the target suggestion box, And use the Soft-NMS algorithm to filter the ROI (Bodla et al., 2017), and finally perform category prediction, bounding box prediction, and target binarization mask for each ROI. The structure of Mask R-CNN is shown in **Figure 4**.

### 3.3 Semantic segmentation model

DeepLabV3+ first uses Xception feature extraction network to perform feature extraction on the original image (Chollet, 2016), and then introduces several parallel Atos convolutions at different rates to obtain larger-scale image feature information. Then use the spatial pyramid pooling module Atrous Spatial Pyramid Pooling(ASPP) (Chen et al., 2016), respectively use a variety of different void rates for extraction. Obtain more semantic feature information, thereby improving segmentation accuracy. The Encode-Decode structure is the mainstream structure in the semantic segmentation network (Badrinarayanan et al., 2015). The so-called encoding process is to extract the features of the substation equipment through the feature extraction network, and then reorganize the feature information through decoding. In this process, the network is based on the image The label information is constantly modified parameters, and finally the object semantic segmentation of supervised learning is realized. The depth separable convolution can be added to the ASPP and decoder modules to make the overall model more efficient.

The UNet proposed by (Ronneberger et al., 2015) for semantic segmentation of biomedical images consists of two stages: a contraction stage and an expansion stage. The shrinking stage consists of the FCN architecture, including convolution, ReLU, and pooling operations. This step is responsible for extracting features from the image. The second step, also known as the expansion step, is the opposite of the previous step. It consists of a series of deconvolution operations followed by convolution and concatenation of the feature maps obtained in the first step. The last part of the network reconstructs the segmented image.

PSPNet (Zhao et al., 2016) adopts a spatial pyramid network architecture, which not only enhances the fusion of multi-scale information, but also reduces the local and global losses. This structure is a network architecture that integrates multi-scale scenarios, including 2 parts of convolutional layer and pyramidal pooling, which has multiple advantages, not only simple architecture but also high flexibility. Among them, the convolutional layer integrates different classical network architectures to achieve a progressive abstraction from low-level to high-level features.

### 3.4 Classification model

**Figure 5:**
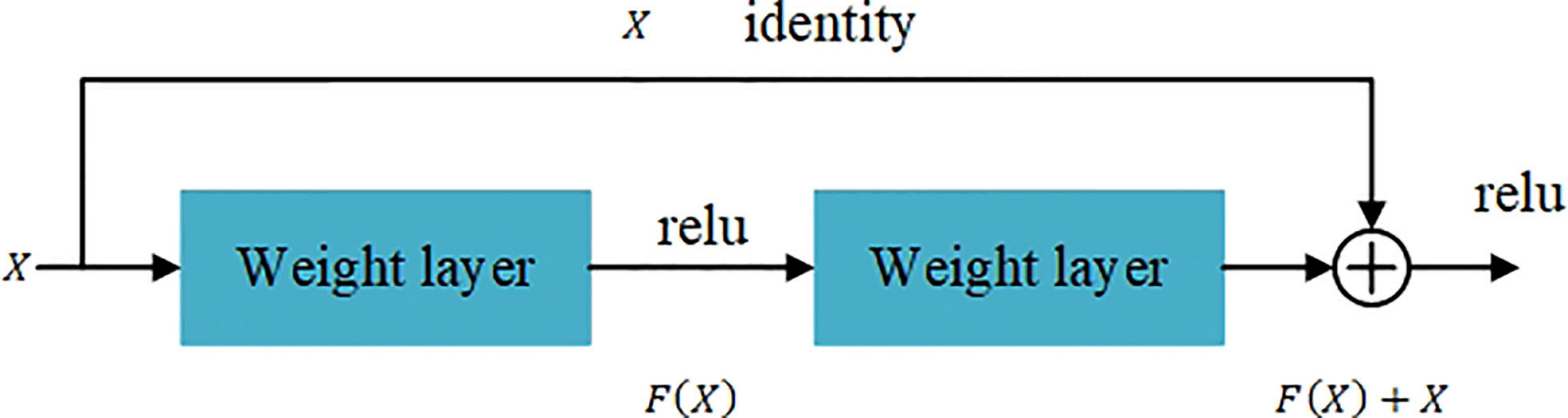
The residual block.

He et al. (2015) proposed resnet50 network. The main contribution is to solve the problem of the decline in classification accuracy as the depth of CNN deepens. The proposed residual learning idea accelerates the CNN training process and effectively avoids the problem of gradient disappearance and gradient explosion. Using the idea of residual learning. He et al. (2015) proposed a Shortcut Connections structure of identity mapping, as shown in **Figure 5**. Where *X* is the input, *F(X)* is the residual mapping, *Y(X)* is the ideal mapping, *Y(X) = F(X) + X*. By transforming the fitted residual mapping *F(X)* into the fitting ideal mapping *Y(X)*, the output can be changed into the superposition of the input and the residual mapping, so that the network changes between the input *X* and the output More sensitive. It does not add additional parameters and calculations to the network, but at *Y(X)* the same time greatly increases the training speed of the model and improves the training effect. When the number of layers of the model is deepened, this simple structure can well solve the degradation problem. In recent years, the ResNet network has been widely cited in various computer vision tasks, and has achieved outstanding performance. So this article chooses ResNet50 as the third stage hierarchical network model.

The essence of the VGG16 model is an enhanced version of the AlexNet structure, with an emphasis on the depth of the CNN design (Simonyan and Zisserman, 2014). Furthermore, each convolutional layer is followed by a pooling layer. VGG16 has five convolutional layers, each with two or three convolutional layers. To better extract feature information, this experiment uses three convolutional layers per segment. In addition, VGG16 uses 3x3 convolution kernels instead of 7x7 convolution kernels. The 3x3 convolution kernel is the smallest receptive field size that can feel the focus of up and down, left and right. And, 2 3x3 convolution kernels are stacked. Their receptive field is equivalent to a 5x5 convolution kernel. When 3 stacks, their receptive field is equivalent to a 7x7 effect. Since the receptive field is the same, three 3x3 convolutions use three nonlinear activation functions to increase the nonlinear expression ability. Makes the dividing plane more separable. At the same time, a small convolution kernel is used, which greatly reduces the amount of parameters. Using the 3x3 convolution kernel stacking form not only increases the number of network layers but also reduces the amount of parameters. Due to the large number of layers and the relatively small convolution kernel, the entire network has better feature extraction effect.

MobileNetV3 replaces part of the 3×3 depth wise convolution by introducing a 5×5 depth wise convolution (Howard et al., 2019). Introduce Squeeze-and-excitation (SE) module and h-swish (HS) activation function to improve model accuracy. The last two layers of pointwise convolution do not use batch normalization. Use the NBN logo in the MobileNetV3 structure diagram. MobileNetV3 combines the following advantages. The first point is the depth wise separable convolution of MobileNetV1. The second point is the inverse residual structure of MobileNetV2 with a linear bottleneck. The third point is to use h-swish instead of the swish function.

### 3.5 Three-stage model structure

Different instance segmentation, semantic segmentation and classification models have different network structures, which will affect the classification accuracy of ‘Huangguan’ pear and disease. If the same model is used in the three stages, the feature extraction ability of the model may be affected due to different segmentation targets. Therefore, based on the different features to be extracted at each stage, compare various semantic segmentation and hierarchical models to determine a better model for each stage. Then, combined with the actual environment’s requirements for segmentation speed, by adjusting the order of the model and changing the feature extraction network, segmentation accuracy can be improved and segmentation time can be shortened.

**Figure 6:**
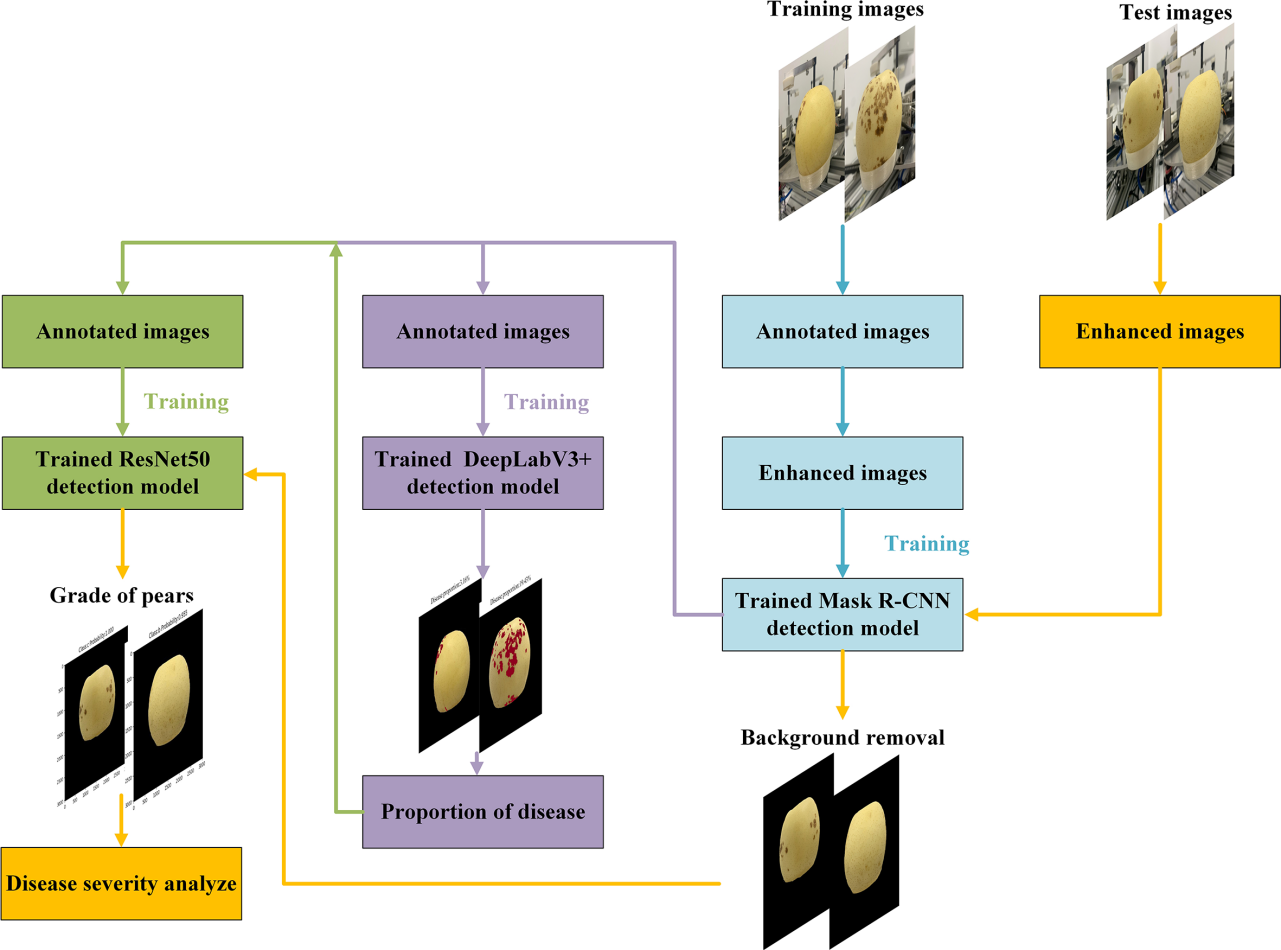
Three-stage model network architecture.

In this research, the fusion instance, semantic segmentation and classification network were used to segment the fruits and lesions of the ‘Huangguan’ pear in two stages through multiple experiments. Because the single-stage segmentation model of ‘Huangguan’ pear in complex background is difficult to accurately segment the fruit and lesions of ‘Huangguan’ pear at the same time, its segmentation accuracy is generally low. Based on the above ideas, through the comparison of multiple instance segmentation models, semantic segmentation models and hierarchical models. Finally, it is determined that the Mask R-CNN network with a preprocessing module is used to segment the ‘crown’ pear in the complex background in the first stage, and the image of the ‘crown’ pear in the simple background can be obtained. Then, the ‘Huangguan’ pear was segmented by DeepLabV3+, and the disease rate of the ‘Huangguan’ pear was calculated. Finally, use ResNet50 for training. The overall flow chart is shown in **Figure 6**.

## 4. Test results and analysis

### 4.1 Accuracy and effect of ‘Huangguan’ pear background segmentation

**Figure 7:**
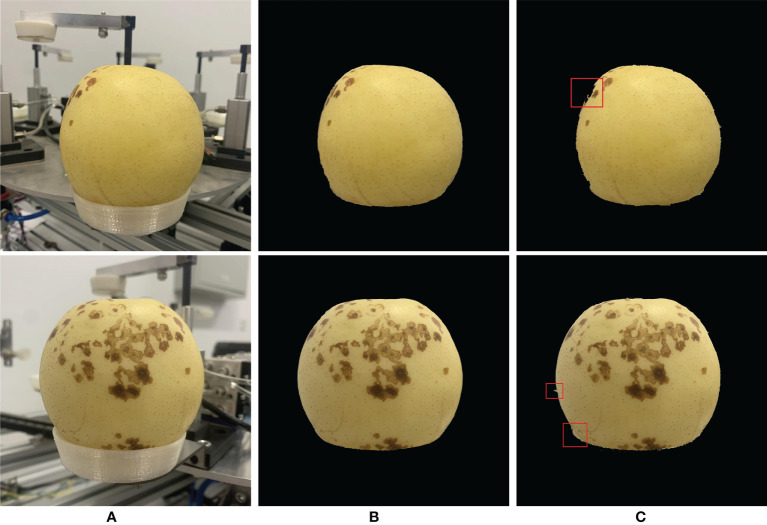
The prediction result of ‘Huangguan’ pear instance segmentation. **(A)** Original images, **(B)** CLAHE-Mask R-CNN and **(C)** Mask R-CNN.

The models used in the first stage of this article are CLAHE-MASK R-CNN and Mask R-CNN. By default, the file with the best training effect will be saved as a weight file and then used for testing. In the algorithm of this paper, the CLAHE preprocessing module is added according to the characteristics of the ‘Huangguan’ pear background, which improves the local contrast of the edge of the ‘Huangguan’ pear and improves the network’s ability to predict the details of the mask boundary. At the same time, fewer convolutional layers can ensure that the edge of the ‘Huangguan’ pear target will not be lost after multi-layer convolution. It can be seen from **Figure 7**. That CLAHE-Mask R-CNN can segment the background other than ‘Huangguan’ pear under the same label picture. Under the same conditions, when the segmented background color is similar to ‘Huangguan’ pear, Mask R-CNN gets the wrong result. The red box marks the background that Mask R-CNN has not segmented completely or the background is segmented excessively. In this paper, the Mask R-CNN of the CLAHE module has a better effect on the edge segmentation of ‘Huangguan’ pear. If the accuracy of the first stage segmentation is not high, it may result in segmentation of the wrong ‘Huangguan’ pear in the second stage, and the final accuracy will be reduced. For comprehensive comparison, CLAHE-Mask R-CNN is selected as the first stage segmentation model. It can be seen from **Table 1** that in the first stage, the segmentation accuracy of Mask R-CNN with the addition of the CLAHE module is significantly higher than that of Mask R-CNN. The PA of CLAHE-Mask R-CNN reaches 97.38%, which can better provide ‘Huangguan’ pear pictures with complex background removed for the next stage and increase the accuracy of the overall model.

**Table 1:** Performance of the first stage model on the test set.

Model	PA/%	Dice/%	IoU/%
CLAHE-Mask R-CNN	97.38	68.08	73.25
Mask R-CNN	94.84	67.72	69.92

### 4.2 Comparison of segmentation accuracy and effect of ‘Huangguan’ pear disease

**Figure 8:**
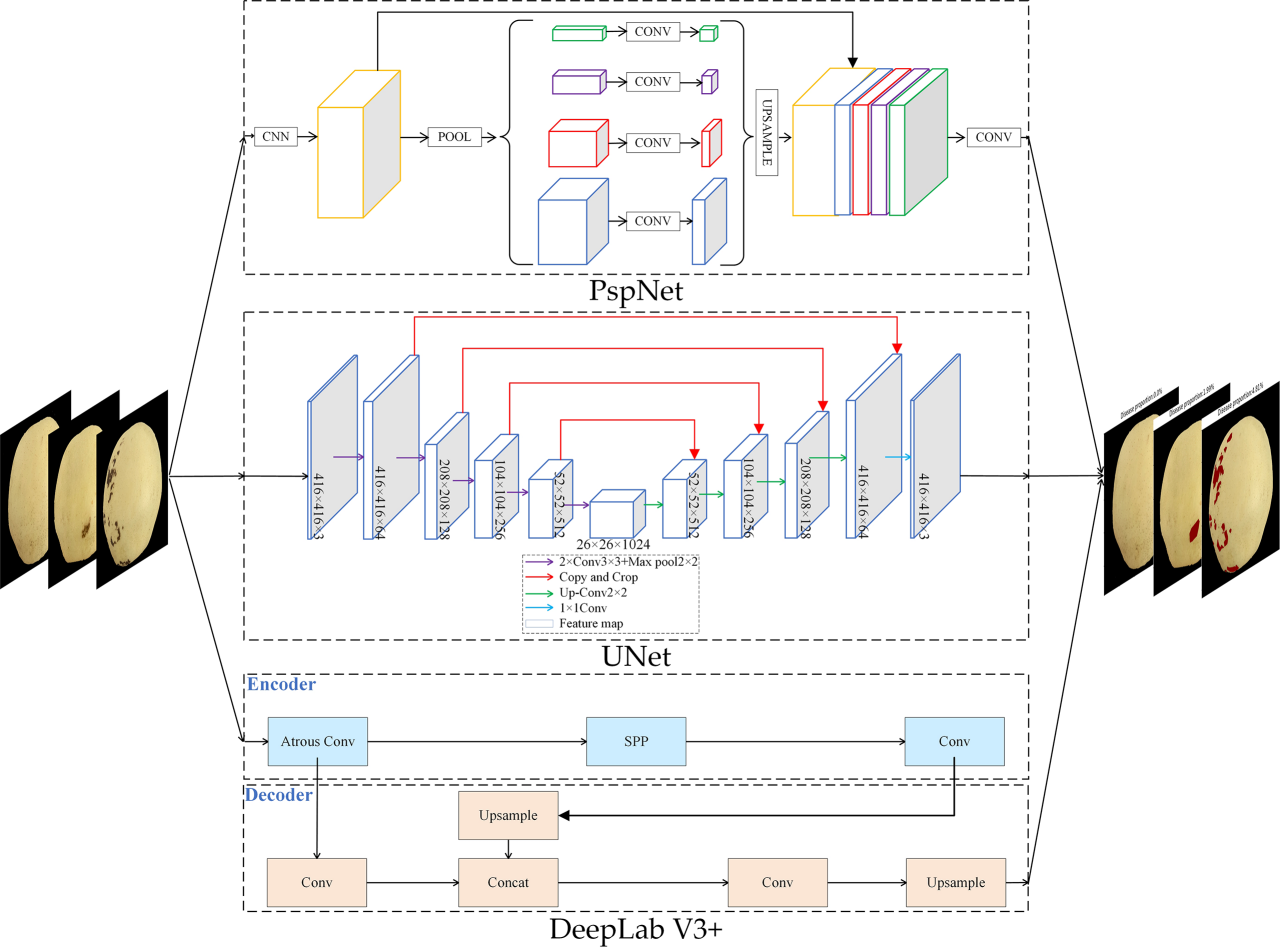
‘Huangguan’ pear semantic segmentation network structure diagram.

The overall structure of the semantic segmentation model used in the second stage is shown in **Figure 8**. Three models DeepLabV3+, UNet, and PspNet were used to segment ‘Huangguan’ pear disease. Divide the area of ‘Huangguan’ pear diseased spots by the area of ‘Huangguan’ pear to get the proportion of diseased spots, which provides accurate data support for the third-step classification model.

**Figure 9:**
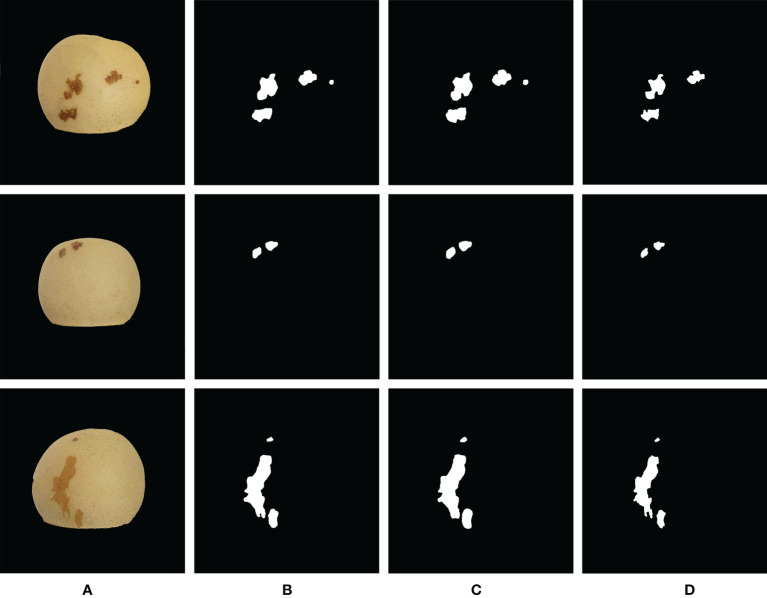
Comparison of the segmentation results of ‘Huangguan’ pear disease. **(A)** Original Image, **(B)** DeepLabV3+, **(C)** UNet and **(D)** PspNet.

In the semantic segmentation stage, 448 images of ‘Huangguan’ pear were used as test samples, and the labels were only divided into ‘Huangguan’ pear and diseased spots without considering the disease category. The test result is the average of the test results of 448 images. **Table 2** shows the comparison results of the segmentation accuracy of each algorithm. It can be seen from **Table 2** that the segmentation accuracy of DeepLabV3+ is significantly higher than that of UNet and PspNet. The accuracy of DeepLabV3+ reached 94.03%. Compared with UNet and PspNet, the accuracy has increased by 2.81% and 0.62%. At the same time, the disease segmented by DeepLabV3+ obtained higher Dice coefficient (0.6725) and IoU coefficient (0.7436). Compared with UNet, it increased by 2.68% and 7.21%, and compared with PspNet by 0.86% and 3.25%. Various segmentation results are shown in **Figure 9**.

**Table 2:** Performance of the second stage model on the test set.

Model	PA/%	Dice/%	IoU/%
DeepLabV3+	94.03	67.25	74.36
UNet	91.22	64.57	67.15
PspNet	93.41	66.39	71.11

**Figure 10:**
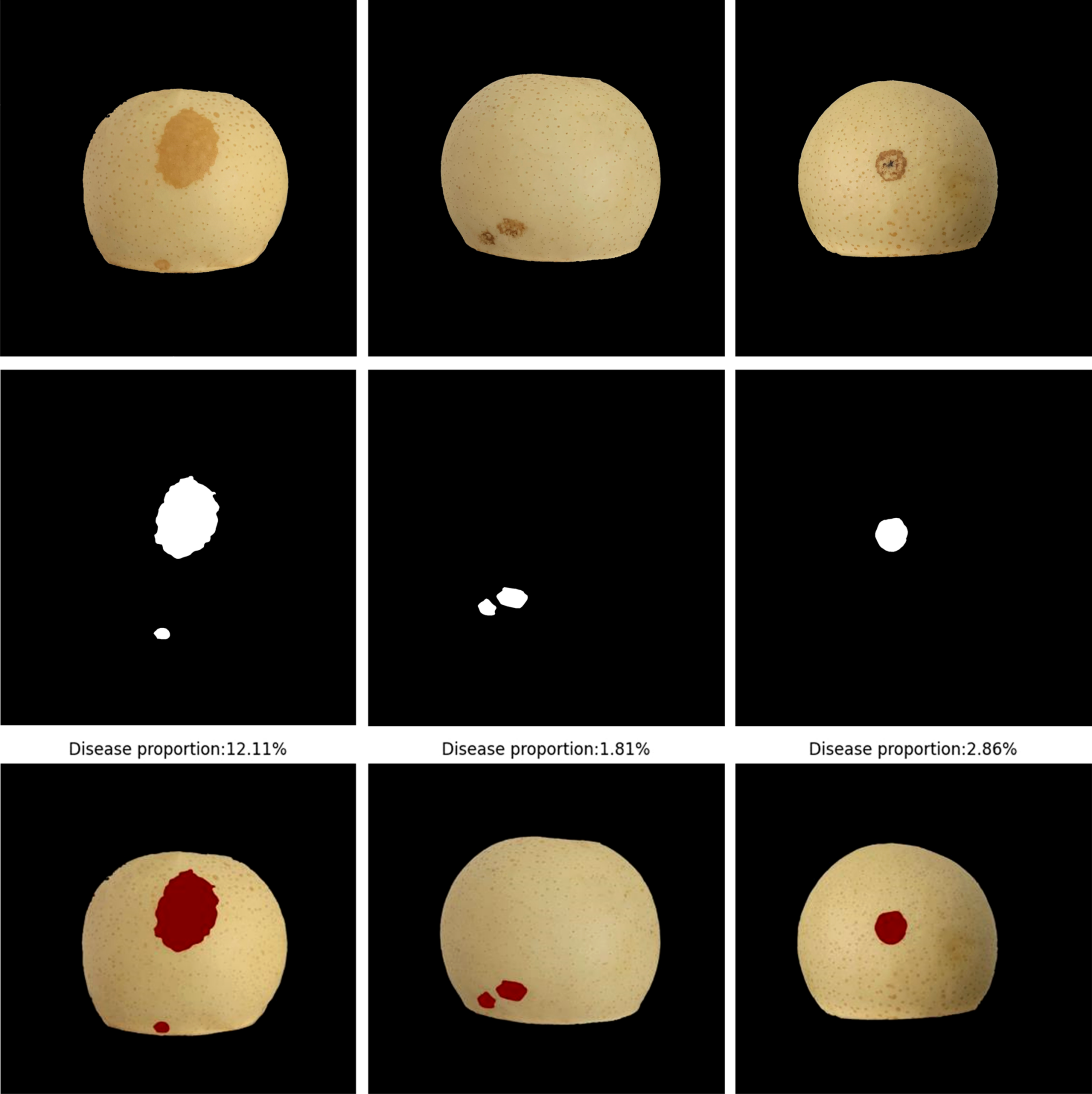
Proportion of ‘Huangguan’ pear disease.

It shows the segmentation results of ‘Huangguan’ pear disease on the DeepLabV3+, UNet, and PspNet models. It can be seen that DeepLabV3+ can segment small disease. The segmentation result of UNet will lose some details, the segmentation boundary will be fuzzy, the similar disease area will be stuck, and the segmentation edge of UNet will appear jagged and there will be edge loss. This is because UNet cannot capture features at different levels, and integrates them through feature superposition. It is easy to lose data due to repeated downsampling and upsampling of the deep network. The convolution operation of the encoder-decoder of DeepLabV3+ can smoothly segment the edges of disease. The segmentation edge of PspNet is relatively smooth, but it is easy to miss some disease areas and excessive segmentation of disease areas. Which means that PspNet does not have obvious response to disease with similar colors to ‘Huangguan’ pear. It can be seen from the segmentation map that the difficulty of segmentation for different disease is different. For example, the chicken feet disease area of ‘Huangguan’ pear is dark yellow and the color is similar to that of ‘Huangguan’ pear, and the edge of the disease is not obvious, so the segmentation is more difficult. DeepLabV3+ can arbitrarily control the resolution of the extracted features of the encoder, and can effectively and accurately segment the ‘Huangguan’ pear disease by balancing the accuracy and time-consuming hole convolution. The proportion of diseased spots in ‘Huangguan’ pears is shown in **Figure 10**.

The DeepLabV3+ model is used to predict the disease area of ‘Huangguan’ pear, and the predicted disease area and the actual disease area have a higher IoU. It benefits from the early pixel-level disease labeling and the introduction of hole convolution in DeepLabV3+, which has strong semantic segmentation performance. According to the ratio of the number of pixels of the diseased spots to the number of pixels of ‘Huangguan’ pear, the accurate ratio of the diseased spots can be obtained, which provides an accurate data set for the third stage model.

### 4.3Analysis of the classification results of ‘Huangguan’ pear

#### 4.3.1 Loss function

The fully connected layer uses the gradient descent algorithm as the parameter optimizer, and sets the average cross entropy as the loss function as follows:


(8) $$L=\frac{1}{N}\Sigma_{i}L_{i}=-\frac{1}{N}\Sigma_{i=1}^{M}y_{i}\ln(p_{i})$$

(8) Where: *N* is the total number of samples; *M* is the number of categories; *y_i_* is the indicator variable (0 or 1), if the category is *i*, it is 1, otherwise it is 0; *p_i_* is the probability that the observed sample is *i*; *L_i_* Represents the loss value of category *i*.

#### 4.3.2 Training process

The data set is classified according to the disease grades segmented by DeepLabV3+. There are a total of 5114 images in the training set and the verification set, which are allocated at a ratio of 9:1. There are 448 images in the test set. When training the classification model, three test models are designed: ResNet50, VGG16 and MobileNetV3. Among them, the classification of ‘Huangguan’ pear image is shown in **Table 3**.

**Table 3:** Grade distribution of ‘Huangguan’ pear.

Dataset split	A	Grade B	C
Training set	1264 (27.46%)	1516 (32.95%)	1822 (39.59%)
Validation set	140 (27.35%)	169 (33.00%)	203 (39.65%)
Test set	126 (28.13%)	130 (29.02%)	192 (42.85%)

Taking the ResNet50 model training as an example, first use a part of the third-class fruits in the image divided into 50 evenly and use equation (9) to train for one round, which can guide the network to pay attention to the disease part when extracting features. Then, after training 30 batches of samples without disease, use the training set with disease for one round to ensure continuous supervision of the results of disease. An epoch training will be completed until all training samples with no disease are finished. At the end of each epoch training, record the training accuracy and average loss. Use the model trained in this round to make a prediction for all test samples, and record the test accuracy and average loss. After training for 150 epochs, the weight with the smallest disease recognition loss on the test set is selected as the final model.

#### 4.3.3 Analysis of training results

**Figure 11:**
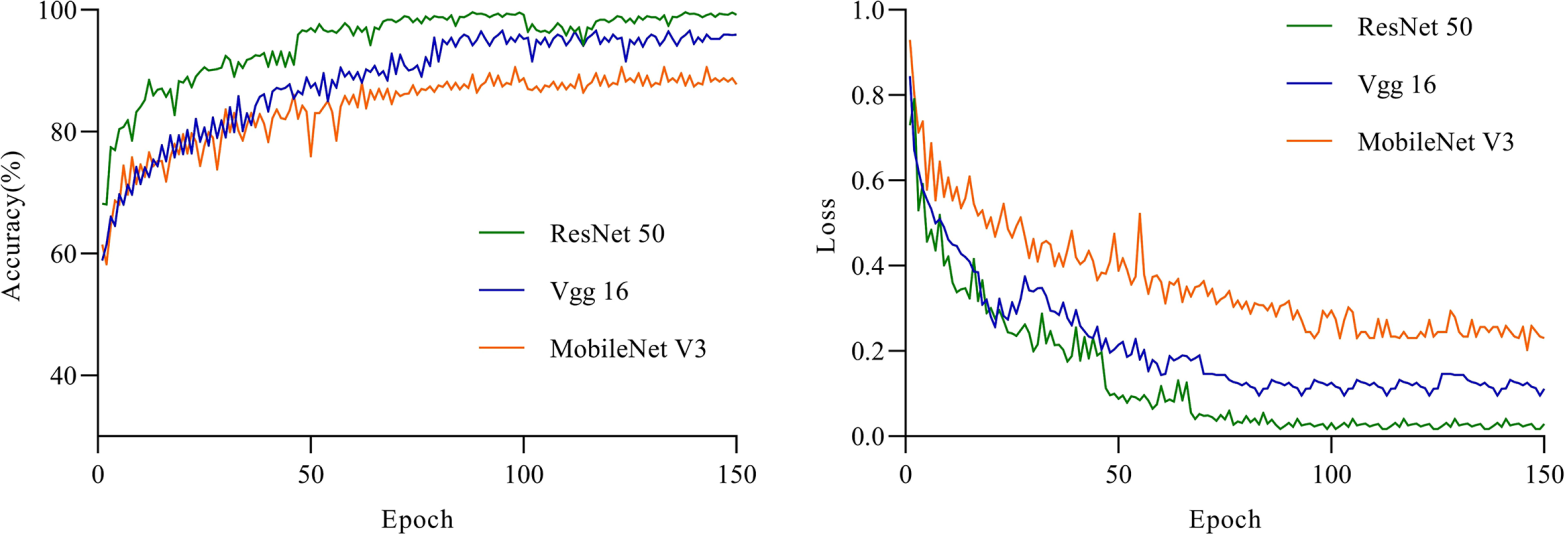
The accuracy and loss of the three models for the test set.

Use VGG16 model, ResNet50 and MobileNetV3 respectively for training, and ensure that the parameter settings are the same. Because each iteration randomly uses an image enhancement method, the training recognition accuracy will fluctuate slightly. In the first 5 rounds of training, the training and recognition accuracy of ResNet50 increased rapidly, and the recognition accuracy on the test set reached more than 95% earlier than other models. ResNet50 has the highest recognition accuracy in the first round of testing. When the number of iteration rounds is about 60 rounds, the training recognition accuracy of ResNet50 first tends to 100%. It can be seen from the change of recognition accuracy that the ResNet50 model can converge faster, and its training accuracy and loss rate are shown in **Figure 11**.

**Figure 12:**
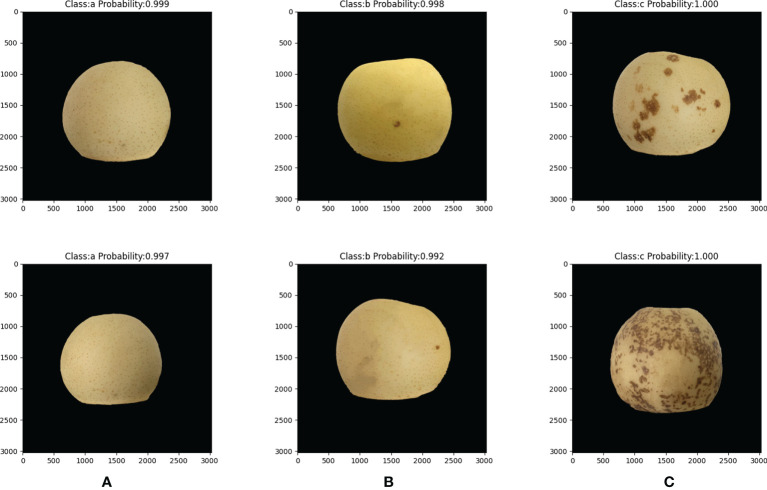
Prediction grade results of ‘Huangguan’ pear. **(A)** grade A, **(B)** grade B and **(C)** grade C.

Accurate and efficient ‘Huangguan’ pear appearance quality classification model is of great significance. The automatic scoring method will alleviate the problem of rural labor shortage. In addition, an accurate grading model will indirectly affect market segments and ensure the reliable and stable quality of ‘Huangguan’ Pear agricultural products. As shown in **Table 4**, the above experimental results clearly show the effectiveness of the ResNet50 model on the ‘Huangguan’ pear appearance quality classification model. The ResNet50 algorithm maintains a fairly high accuracy. The results show that this method can be used to realize the automatic grading of the appearance quality of ‘Huangguan’ pear. In our experiments, the ResNet50 model takes about 311.2 milliseconds to predict the appearance quality of each ‘Huangguan’ pear, and there is not much difference between VGG16 and MobileNetV3. This speed can fully meet the real-time requirements of classification. Compared with VGG16 and MobileNetV3, the average precision of ResNet50 has a higher advantage, which is 11.61% and 4.94% higher respectively. The prediction result of ‘Huangguan’ pear grade is shown in **Figure 12**.

**Table 4:** Comparison results of ‘Huangguan’ pear grading data sets.

Model	Classes	Precision/%	Recall/%	AP/%	F1/%	Speed/ms
ResNet50	A	99.58	97.25	97.41	95.43	311.2
	B	95.26				
	C	97.39				
VGG16	A	88.21	85.54	85.80	86.10	430.6
	B	83.42				
	C	85.78				
MobileNetV3	A	95.97	93.68	92.47	92.88	198.5
	B	89.28				
	C	92.15				

It can be seen that the prediction result of ResNet50 on the appearance quality of ‘Huangguan’ pear is relatively accurate, and the prediction level and prediction probability are marked directly above each picture. It can be seen that the ResNet50 model can predict the ‘Huangguan’ pear images with different light intensity well, and the model has high robustness.

## 5. Conclusion and future work

In conclusion, this research proposes a three-stage model of ‘Huangguan’ pear disease in complex contexts that combines instance segmentation, semantic segmentation, and classification. In the first stage, the complete ‘Huangguan’ pear fruit is segmented and extracted using Mask R-CNN with an preprocessing module. Then in the second stage, DeepLabV3+ was used to segment and extract the diseases of the simple background ‘Huangguan’ pear fruit extracted in the first stage, and the proportion was calculated. Through the data obtained in the second stage, the ‘Huangguan’ pears are divided into three grades: A, B, and C. In the third stage, the weights are obtained by training three grades of fruits through ResNet50. In the prediction stage, after the Mask R-CNN segmentation is completed, the ResNet50 model is used for prediction, and the grade of the ‘Huangguan’ pear can be directly obtained. Overall, the model can improve the accuracy of disease segmentation, thereby providing a reasonable classification opinion for the disease severity of ‘Huangguan’ pear fruits. Finally, the pixel accuracy of the Mask R-CNN model with preprocessing module is 97.38%. The pixel accuracy of the DeepLabV3+ model is 94.03%. The average precision of the ResNet50 model is 97.41%. Overall segmentation and classification performance is significantly improved compared to the one-stage model. This method based on machine vision and deep learning is harmless to ‘Huangguan’ pears and provides technical support for follow-up research. Currently, all diseases are roughly graded. ‘Huangguan’ pears suffer from a wide variety of diseases. The next step will be to subdivide the disease of the ‘Huangguan’ pear. Detect and identify various types of diseases to assess their severity. Thereafter, further work in this direction will continue.

## Data availability statement

The raw data supporting the conclusions of this article will be made available by the authors, without undue reservation.

## Author contributions

YZ: Writing-original draft. NS: Guiding, Supervision. HZ: Data collection. JZ: Proofreading and polish manuscript. XF and XS: Editing, Supervision, Proofreading. All authors contributed to the article and approved the submitted version.

## Funding

This study was supported by the Key Research and Development Program of Hebei Province (20327213D).

## Conflict of interest

The authors declare that the research was conducted in the absence of any commercial or financial relationships that could be construed as a potential conflict of interest.

## Publisher’s note

All claims expressed in this article are solely those of the authors and do not necessarily represent those of their affiliated organizations, or those of the publisher, the editors and the reviewers. Any product that may be evaluated in this article, or claim that may be made by its manufacturer, is not guaranteed or endorsed by the publisher.
